# Converting single nucleotide variants between genome builds: from cautionary tale to solution

**DOI:** 10.1093/bib/bbab069

**Published:** 2021-04-05

**Authors:** Cathal Ormond, Niamh M Ryan, Aiden Corvin, Elizabeth A Heron

**Affiliations:** Neuropsychiatric Genetics Research Group in the Department of Psychiatry, Trinity College Dublin, Ireland; Neuropsychiatric Genetics Research Group in the Department of Psychiatry, Trinity College Dublin, Ireland; Neuropsychiatric Genetics Research Group and the Head of the Department of Psychiatry, Trinity College Dublin, Ireland; Neuropsychiatric Genetics Research Group in the Department of Psychiatry, Trinity College Dublin, Ireland

**Keywords:** genome build conversion, liftOver, CrossMap, GRCh37, GRCh38

## Abstract

Next-generation sequencing studies are dependent on a high-quality reference genome for single nucleotide variant (SNV) calling. Although the two most recent builds of the human genome are widely used, position information is typically not directly comparable between them. Re-alignment gives the most accurate position information, but this procedure is often computationally expensive, and therefore, tools such as *liftOver* and *CrossMap* are used to convert data from one build to another. However, the positions of converted SNVs do not always match SNVs derived from aligned data, and in some instances, SNVs are known to change chromosome when converted. This is a significant problem when compiling sequencing resources or comparing results across studies. Here, we describe a novel algorithm to identify positions that are unstable when converting between human genome reference builds. These positions are detected independent of the conversion tools and are determined by the chain files, which provide a mapping of contiguous positions from one build to another. We also provide the list of unstable positions for converting between the two most commonly used builds GRCh37 and GRCh38. Pre-excluding SNVs at these positions, prior to conversion, results in SNVs that are stable to conversion. This simple procedure gives the same final list of stable SNVs as applying the algorithm and subsequently removing variants at unstable positions. This work highlights the care that must be taken when converting SNVs between genome builds and provides a simple method for ensuring higher confidence converted data. Unstable positions and algorithm code, available at https://github.com/cathaloruaidh/genomeBuildConversion

## INTRODUCTION

The human reference genome is a fundamental and essential resource for next-generation sequencing studies, aiding in tasks such as genome assembly and variant calling. Without a reference, *de novo* assembly of each sequenced genome would need to take place, which is computationally intensive and in certain scenarios may result in a poor quality assembly [1]. The most frequently used human reference genomes are those constructed by the Genome Reference Consortium (GRC) [2], who to date have released 38 iterative reference builds. The two most recent builds of the genome are GRCh37 (released in 2009) and GRCh38 (released in 2013). The UCSC Genomics Institute have also released analogous versions of these builds, referred to as hg19 and hg38, respectively [3].

Both GRCh37 and GRCh38 were generated by sequencing DNA from a collection of human donors, predominantly using Sanger sequencing [4, 5]. DNA sequences were combined to form high-confidence contiguous segments known as contigs, which were joined to form a *de novo* assembly of the reference genome. One of the major updates in GRCh38 was the closing of numerous gaps where sequencing had previously not been possible [6]. GRCh38 also contains a much larger collection of unlocalized (known sequence and chromosome but position unknown) and unplaced (known sequence, but chromosome and position unknown) contigs, as well as alternate contigs (known alternate representations of specific regions of the genome to account for population differences) [6]. Users need to be aware that different builds result in different genome assemblies and subsequently can impact genomic analyses, including single nucleotide variant (SNV) analyses [7].

Despite the improvements that the latest build brings, updates to the base-pair coordinates typically mean that not all positions are comparable between builds. Researchers are sometimes hesitant to switch to GRCh38, as there exists a wealth of annotation information available for GRCh37 and many pipelines and tools are still based on the older, GRCh37, version [7]. A similar problem arises when comparing new sequencing data to data aligned to an older build, as both data sets must be aligned to the same build to be comparable. Although re-alignment of the original sequence data to the new build typically provides the most accurate base-pair position information, this can be quite computationally expensive [7]. Also, the raw sequence data required for alignment, if available, can be large, so long-term storage may not be feasible. An alternative approach to re-alignment is to convert between genome builds using tools such as *liftOver* (provided as part of the Genome Browser tool [3] hosted by the UCSC Genomics Institute), *CrossMap* [8] or *Remap* (hosted by the National Centre for Biotechnology Information [9]). This process is aided by a chain file, which provides a mapping of contiguous positions from one build to another. The ability to convert between builds using these tools has proved vital, allowing the integration of a wide range of SNV annotation databases and sequence data, regardless of how they were originally aligned, for example gnomAD [10], CADD [11] and dbNSFP [12].

For those who do choose to convert between GRCh37 and GRCh38, there are known problems with this conversion process, particularly for SNVs. In the online user guide for the UCSC Genome Browser, the authors note that ‘occasionally, a chunk of sequence may be moved to an entirely different chromosome’ (see Web resources in Methods section). This is echoed in Liu *et al.* [12], where the authors note that after converting the dbNSFP database to other builds using *liftOver*, ‘there are a few SNVs whose coordinates in hg38 and hg19 … have inconsistent chromosome numbers’. This phenomenon can prove problematic for downstream analyses if, for example, annotation information from converted data is not consistent with annotation information from re-aligned data. For example, suppose we wish to examine variants in protein coding regions of the genome, prioritized using the CADD deleteriousness score [11]. Consider the T > A substitution at position 15690247 on chromosome 22 of GRCh38 (chr22:c.15690247 T > A), contained in the first exon of the *POTEH* gene. CADD v1.6 gives the variant a C-score of 20.8, indicating that it is in the top percentile of all ranked deleterious variants. If we convert the position to GRCh37 (using either *liftOver* or *CrossMap*), this variant maps to position 19553586 on chromosome 14, where the reference allele is still T (chr14:c.19553586 T > A) but the variant is now in the first exon of *POTEG*. CADD v1.6 for GRCh37 gives this variant a C-score of 0.009, indicating that it is now in the bottom percentile of all ranked deleterious variants in the genome. This inconsistency shows how downstream results can be negatively impacted by instabilities in the conversion process.

Pan *et al.* (2019) [13] examined SNVs from data aligned under a range of bioinformatics pipelines to data converted between GRCh37 and GRCh38 using both *liftOver* and *CrossMap*. The authors noted that on average, 1% of SNVs did not convert from GRCh37 to GRCh38, and an average of 5% of SNVs did not convert from GRCh38 to GRCh37. Furthermore, on average, 1.5% of SNVs which were successfully converted were not found in the corresponding aligned data, a trend that was more pronounced when converting from GRCh38 to GRCh37. Such discordant sites were noted to be low-confidence calls, have lower average read depth and have a higher than average GC content. The authors urged caution when converting SNVs between builds.

Recently, Luu *et al.* (2020) [14] benchmarked six tools (including *liftOver*, *CrossMap* and *Remap*) for converting multi-base-pair regions derived from epigenetic data from GRCh37 to GRCh38. The authors found a high degree of correlation between the six tools but noted that gapped regions in both chain files can result in conversion failure, or even regions mapping to incorrect locations. A guideline to improve conversion is offered, which involves removing input data which overlap with the gapped regions, as well as removing input data which map to multiple regions or alternate contigs. However, if this strategy were applied to SNV data, some variants (such as those in un-gapped regions which also change chromosome under conversion) may not necessarily be removed.

Here, we present a novel algorithm to identify base-pair positions in the human genome which exhibit unstable behaviour when converting between genome reference builds. In addition, we are providing the list of these unstable positions for the two most recent builds (GRCh37 and GRCh38). This list can be used to pre-exclude SNVs prior to conversion to remove potentially problematic variants, resulting in stable SNVs and improving the quality of sequencing data post-conversion.

## METHODS

### Full-genome data

As genome build conversion tools are primarily based on base-pair position information only, it is possible to examine all base-pair positions in the genome. This allows the behaviour of all potential SNVs to be examined when converting between builds, rather than just a subset that might be found on an individual sample’s genome. To this effect, a BED entry was created for each base-pair position in both the GRCh37 (GCA_000001405.1) and GRCh38 (GCA_000001405.15) reference genomes, which we refer to as the full-genome data. This includes positions that are not typically amenable to short-read whole genome sequencing (WGS), such as known gaps in the genome assembly. Positions on the unplaced, unlocalized and alternate contigs were not included in the input data, and so only the standard 23 pairs of chromosomes were considered. Each entry was given a label containing the original chromosome and start position for unique identification, and the input BED file was split by chromosome for parallelization [15]. This generated 3 095 677 412 positions for GRCh37 and 3 088 269 832 positions for GRCh38.

### Algorithm to identify novel conversion-unstable positions

**Figure 1:**
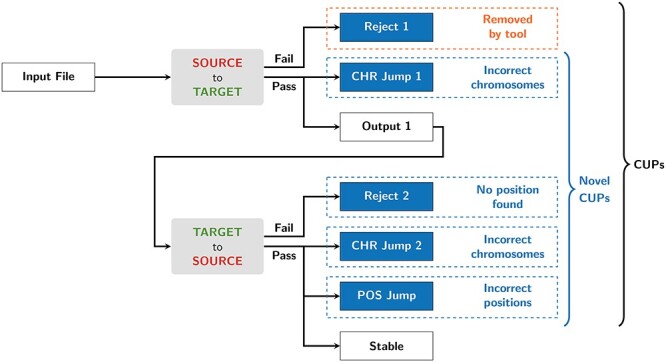
Flow chart of the algorithm to identify novel CUPs.

To identify base-pair positions that are unstable in the conversion process (defined below), each input file was converted from the source build to the target build and then back to the source build again (Figure 1). Entries in the output files were extracted if they satisfied one of the following conditions:

- positions which failed on the first conversion (Reject_1),
- positions which mapped to a different chromosome on the first conversion (CHR_Jump_1),
- positions which failed on the second conversion (Reject_2),
- positions which did not map back to the original chromosome on the second conversion (CHR_Jump_2) and
- positions which did not map back to the original position on the second conversion (POS_Jump).

We refer to these collectively as conversion-unstable positions (CUPs), and all other positions are referred to as stable. Note that entries in the Reject_1 category are typically identified by the conversion tool, so the latter four entries are what we refer to from here on collectively as novel CUPs. Reject_1 and CHR_Jump_1 positions were removed prior to the second conversion (from the target build back to the source build). Despite not being included in the input data, entries that mapped to the unplaced, unlocalized, and alternate contigs were retained in the CHR_Jump_1 and CHR_Jump_2 categories to ensure that each base-pair position had an accurate category designation.

Both *liftOver* and *CrossMap* (v0.4.2) were used for the conversion. *Remap* was not considered as its input file is limited to 250 000 entries, which is much smaller than the lengths of the input chromosomes. Integral to this conversion process is a build-specific chain file, allowing for small-scale differences, e.g. discrepancies arising from fixing base-pair position errors between builds. Chain files mapping between GRCh37 and GRCh38 (one for each direction) were obtained from the *liftOver* website hosted by the UCSC Genomics Institute (see Web resources in Methods section) and the same chain files are used by both *liftOver* and *CrossMap*, allowing us to also check the robustness of CUP identification, as a consensus between tools would give higher confidence in the output. This algorithm was run twice, once for the GRCh37 build as the source and once for the GRCh38 build as the source.

### Comparison with assembly annotation sets

To better understand the possible reasons for CUPs occurring, we also identified where these positions originated. Given the reconstruction of some contigs in the development of GRCh38 [6], one explanation for base-pair positions being rejected during a conversion is that the position is not in the target build. In the online support forum for the UCSC Genome Browser, it is noted that variants may change chromosomes between builds because they lie in repetitive regions or segmental duplications (see Web resources in Methods section). In an attempt to isolate the source of each CUP, the following assembly annotation sets were obtained from the UCSC Table browser [3] for both genome builds:

- Gaps in the build (gap): regions that are not present in the build, including telomeres, the short arms of specific chromosomes and gaps between known contigs. The centromeres are present in the GRCh37 gap set (as they did not form part of the assembly) but are not in the GRCh38 gap set. In the interests of a fairer comparison, the centromeres were removed from the GRCh37 gap set prior to comparison.
- Differences between contigs (hg38ContigDiff): regions that are different in the GRCh38 and GRCh37 builds due to updates in individual contigs.
- Segmental duplications (genomicSuperDups): regions longer than 1 kb that have a high degree of similarity with other regions.

Given the overlap between these sets, positions unique to each of the three sets, as well as positions which were present in more than one set (multiple) or no set (other), were considered (Supplementary Figure S1, Supplementary Data available online at http://bib.oxfordjournals.org/). For the CUPs identified above, contiguous entries were collapsed into multi-base-pair regions using *bedtools* [16], to allow for quicker comparison with the assembly annotation sets. The proportion of overlap in CUP category A of assembly annotation set B is defined as $ |A \cap B| / |A| $ and was computed using *bedtools*.

### Whole genome sequence data

The well-characterized NA12877 and NA12878 samples for the Coriell Institute CEPH 1463 family were used to examine the behaviour of SNVs from WGS data when converting between builds. High-confidence, pedigree-validated variant calls for both samples were obtained from the Illumina Platinum Genomics project in VCF format on both GRCh37 and GRCh38 [17]. As we are only considering the behaviour of SNVs and aim to compare the WGS data with the full-genome data, only biallelic SNVs were extracted for both samples. A slightly modified version of the above algorithm was implemented using the *LiftoverVcf* module from *picard* rather than *liftOver*, as *liftOver* does not handle VCF file format. *CrossMap* can accommodate VCF file format. The *LiftoverVcf* module is based on *liftOver* but additionally checks the reference allele of each variant with the target reference genome, removing any sites where there is a mismatch. For VCF files, *CrossMap* updates the reference allele to that of the target build where there is a discrepancy and returns a failure if the alternate allele on the source build is the same as the updated reference allele on the target build. If a reference allele was updated to an ambiguous base (denoted by IUPAC codes), these were removed and considered a mismatch. For the WGS data, two additional output categories were included for variants which failed due to reference-allele mismatches on the first conversion (Mismatch_1) or on the second conversion (Mismatch_2). Position and genotype discordance rates between the converted and the aligned data were computed using *bedtools* and *GenotypeConcordance* (from *picard*), respectively. These were calculated as the proportion of variants in the converted data where the position/genotype did not match that of a variant in the aligned data. Genotype discordance rates are calculated as a proportion of variants whose position matched a variant in the aligned data.

Since individual base-pair positions are converted independently of one another, variants, which are present in any of the novel CUPs can also be excluded prior to conversion to ensure all variants, are stable and data are of high quality. These filtered data were compared with the output from the algorithm on the original data to confirm that both methods are equivalent. In addition to the VCF data files, BED files were generated using position information extracted from the VCF data. This allowed us to apply our original position-based algorithm (that used the *liftOver* and *CrossMap* tools) for comparison.

### Web resources

- UCSC Genome Browser user guide on build conversion: https://genome.ucsc.edu/goldenPath/help/hgTracksHelp.html#Convert
- UCSC Genome Browser support forum on *liftOver* errors, with variants swapping chromosomes: https://groups.google.com/a/soe.ucsc.edu/g/genome/c/P3M1Q5baozM/m/Slyjdco5BwAJ
- The online implementation of *liftOver*: https://genome.ucsc.edu/cgi-bin/hgLiftOver
- The online manual for *CrossMap*: https://crossmap.readthedocs.io/en/latest/
- Chain files for GRCh37 to GRCh38, provided by the UCSC Genomics Institute: http://hgdownload.cse.ucsc.edu/goldenpath/hg19/liftOver/hg19ToHg38.over.chain.gz
- Chain files for GRCh38 to GRCh37, provided by the UCSC Genomics Institute: http://hgdownload.cse.ucsc.edu/goldenpath/hg38/liftOver/hg38ToHg19.over.chain.gz
- Illumina Platinum Genomes project: https://www.illumina.com/platinumgenomes.html

## RESULTS

### Full-genome data

We examined every base-pair position in both builds of the human reference genome to identify positions that are unstable to conversion. Both *liftOver* and *CrossMap* gave identical output for the same input data (Table 1; Supplementary Table S1, Supplementary Data available online at http://bib.oxfordjournals.org/). On GRCh37, ~11.3 Mb of novel CUPs were identified (representing 0.37% of the build), and on GRCh38, ~20 Mb of novel CUPs were identified (0.65% of the build). For both builds, a successive application of the algorithm on the stable positions using either tool did not identify any additional base-pair positions for any of the CUP categories.

**Table 1:** Details of the stable positions and CUPs for the full-genome data for GRCh37 and GRCh38, including the number of base-pairs (bps) for each category and the proportion of the genome build covered (%). Novel CUPs are highlighted in grey

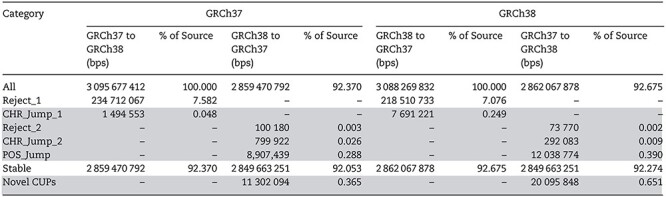

**Figure 2:**
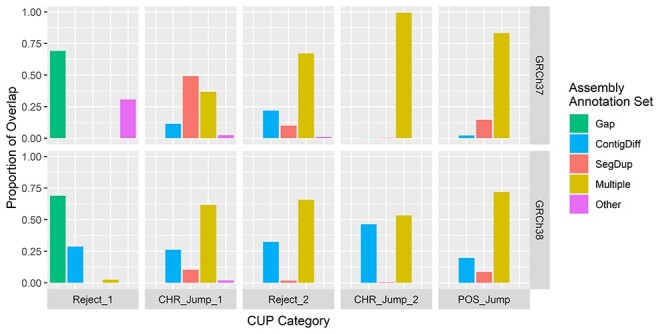
The proportion of CUPs that overlaps with the assembly annotation sets, for the GRCh37 (top) and GRCh38 (bottom) builds. Here, ‘Multiple’ represents positions present in one or more of the assembly annotation sets and ‘Other’ represents positions present in none of the assembly annotation sets (this includes the centromeres for GRCh37). Gap: gaps in the assembly; ContigDiff: differences in contigs between builds; SegDup: segmental duplications.

We compared each of the CUPs with three assembly annotation sets (gaps in the assembly, contig differences and segmental duplications). For both builds, the proportion of overlap for each CUP category across all the assembly annotation sets was at least 97.5% for all except the Reject_1 category on GRCh37, where the proportion was 69.2% (Figure 2). However, the centromeres that were removed from the gap set (which do not overlap with the other assembly annotation sets) account for an additional 29.4% of the Reject_1 category, giving a total overlap proportion explained of 98.6% (Supplementary Table S2, Supplementary Data available online at http://bib.oxfordjournals.org/). A large proportion (~70%) of Reject_1 categories on both builds are composed of the gap set, whereas the novel CUPs are heavily dominated by the contig differences and segmental duplications.

### WGS data

As a proof of principle, we also examined the presence of CUPs in WGS data for two individuals from the CEPH 1463 family. Sample NA12877 had 3 518 008 SNVs on GRCh37 and 3 576 396 SNVs on GRCh38. Sample NA12878 had 3 523 638 SNVs on GRCh37 and 3 594 064 SNVs on GRCh38. Each of these represents ~0.1% of the full genome data for their respective build. For both samples, the CUPs identified from the VCF data were contained within the CUPs identified from the corresponding BED data, as expected. The only positions from the VCF data that were not contained in the BED data were the mismatch categories (due to reference allele mismatches). Furthermore, the CUPs identified from the BED positions from the WGS data were contained within the respective full-genome CUPs. *liftOver* and *CrossMap* broadly agreed on the CUPs derived from the VCF data, with differences arising purely due to how each tool treats the reference allele in the target build, including ambiguous bases (Mismatch_1, Mismatch_2).

The number of stable SNVs was the same for the filtered data (novel CUPs excluded) as for the unfiltered original WGS data when the algorithm was applied (Table 2; Supplementary Tables S3 and S4, Supplementary Data available online at http://bib.oxfordjournals.org/). As expected, no additional variants in the CUP categories were identified on a successive application of the algorithm to either the original data or to the filtered data. The SNVs at novel CUPs represented ~0.13% of SNVs on either build. The position and genotype discordance metrics between the converted and aligned data are given in Supplementary Table S5, Supplementary Data available online at http://bib.oxfordjournals.org/.

**Table 2:** Counts of base-pair positions (bps) and proportions (%) of all SNVs present in WGS data for sample NA12878 broken down by genome build (GRCh37, GRCh38), conversion tool (*liftOver* or *CrossMap*) and whether the original or filtered data were considered

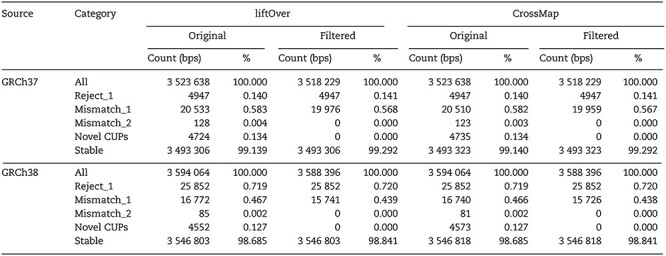

All novel CUPs have been combined into one entry in the table (novel CUPs, highlighted in grey), see Supplementary Tables S3 and S4, Supplementary Data available online at http://bib.oxfordjournals.org/, for a full breakdown of the individual novel CUP categories.

## DISCUSSION

Here, we have replicated the previously observed phenomenon whereby a small proportion of SNVs change chromosome when they are converted to another genome build [12]. Additionally, we have identified novel sites where base-pair position information does not behave as expected or where a one-to-one mapping between positions on both builds is not present. The novel CUPs represent 0.37% of the GRCh37 build and 0.65% of the GRCh38 build. This is important, as annotation data rely heavily on position information and downstream analysis can be negatively impacted by inaccuracies during the conversion process, as evidenced by our motivating example above.

The CUPs show a high degree of overlap with the three assembly annotation sets. For both builds, the Reject_1 positions (failure of the first conversion between builds) are dominated by the gap and contig differences sets. This is a highly plausible explanation for these base-pair positions as the conversion tools will fail when regions of the genome are not present, or have been updated, in the target build. For example, on GRCh37, the centromeres make up ~30% of the Reject_1 category (appearing in the ‘Other’ set in Figure 2), which is to be expected as the centromeres were broadly reconstructed during the assembly of GRCh38. The intersection between the contig differences and segmental duplications accounts for less than 6% of all the assembly annotation sets (Supplementary Figure S1, Supplementary Data available online at http://bib.oxfordjournals.org/); however, the novel CUPs are largely composed of this intersection. If a region is contained in both a segmental duplication and a contig difference, this may indicate that the region is better placed in another part of the genome, which would explain the conversion instability. There remains a small proportion of each of the unstable regions that is not covered by at least one of the three assembly annotation sets (Figure 2, Supplementary Table S2, Supplementary Data available online at http://bib.oxfordjournals.org/).

In our study, both conversion tools identified the same unstable regions, which accords with the findings of Luu *et al.* 2020 [14], in their study of six conversion tools (including *liftOver* and *CrossMap*). Additionally, once the novel CUPs are removed from the full-genome data, a successive application of the algorithm on the stable positions does not identify any further novel CUPs, meaning that there is a one-to-one mapping for all stable base-pair positions between builds. Finally, the WGS data fully agree with the theoretical full-genome data. The comparison between filtered and original WGS data shows that pre-excluding variants at novel CUPs results in the same list of stable variants as applying the full-genome algorithm to the original WGS data. We provide a list of regions to exclude so that the user may remove any variants in novel CUPs prior to conversion.

Pan *et al.* 2019 [13] reported conversion failure rates for WGS data of on average 1% from GRCh37 to GRCh38 and 5% from GRCh38 to GRCh37, noting that the SNVs that failed tended to have much lower depth of coverage and may represent false-positive variant calls. Here, we observe much lower tool conversion failure rates of 0.14% from GRCh37 to GRCh38 and 0.72% from GRCh38 to GRCh37 for the WGS data. We note that the SNVs used in the analysis here were detected by multiple calling algorithms and have been pedigree-validated by confirming a Mendelian inheritance pattern in the samples' children, suggesting that this dataset is a particularly clean and accurate set of SNVs [17]. This may account for the decrease in conversion failure rates compared with the previous study. However, we note that the trend in performance is in the same direction and that converting from GRCh37 to GRCh38 is more accurate than GRCh38 to GRCh37. While Pan *et al.* (2019) show that read depth and variant quality may have an impact on discordance rates, the variants examined here did not have this information available, and thus, we were unfortunately not able to assess these aspects of the novel CUPs.

The combined position and genotype discordance rates were on average 3.07% when converting from GRCh38 to GRCh37 and 1.68% when converting from GRCh37 to GRCh38 (Supplementary Table S5, Supplementary Data available online at http://bib.oxfordjournals.org/). When variants in the novel CUPs were pre-excluded, these rates reduced to 2.97 and 1.61%, respectively. This is higher than the average discordance rate observed by Pan *et al.* (2019) of 1.5%; however, these rates are not directly comparable. Pan *et al.*’s average discordance rate is taken across all bioinformatics pipelines, across both builds and across both tools. Although Pan *et al.* (2019) do not provide the exact rates to compare, our discordance rates are broadly in line with those observed in their Figure 6A [13]. As with the conversion failure rates, both this study and Pan *et al.* (2019) found that converting from GRCh38 to GRCh37 yields higher discordance rates. We note that the genotype discordance rates are quite low at an average of 0.0011% for both builds (Supplementary Table S5, Supplementary Data available online at http://bib.oxfordjournals.org/). This indicates that when the position of a variant has been correctly converted, the genotype is also highly likely to be correct.

This study has some limitations. Firstly, since two independent conversion tools generate identical CUPs, we conclude that these regions are determined by the chain files as both tools utilize the same chain files. This is important to note, as alternative chain files exist for converting between GRCh37 and GRCh38, and therefore, the full algorithm will need to be applied if different chain files are used. A link to the source code used to generate the CUPs is provided for this purpose. However, it is worth noting that the chain files used here are the only ones supplied by the authors of *liftOver* and *CrossMap*. Secondly, while the full-genome data give insight into the behaviour of SNVs under build conversion, this does not account for regions spanning multiple base-pairs, as conversion tools are typically sensitive to this [14]. Finally, we have used aligned WGS data as a gold standard for evaluating the accuracy of converted data, but it is important to note that although the SNVs are pedigree-validated, they may still contain false positive variant calls.

Here, we have clearly highlighted the care that must be taken when converting between genome builds to ensure high-quality data. Although we have shown results for the two most recent builds of the human genome, the same argument can be applied when converting between any other build pair, or indeed for non-human genomes. Unless the user is familiar with the instabilities we have described, we recommend following the simple strategy devised here of removing variants at novel CUPs to ensure high-confidence data when converting SNVs between the two most recent builds of the human genome.

**Key Points**

- When using tools such as *liftOver* and *CrossMap* to convert SNVs between the two most recent builds of the human reference genome (GRCh37 and GRCh38), some base-pair positions map to different chromosomes.
- Additionally, when converting from target build back to the source build, there are base-pair positions which do not map back to the same original position. This means that for these base-pair positions, a one-to-one correspondence between builds does not exist.
- These CUPs are predominantly comprised of regions with known annotation: gaps in the assembly, contig differences between builds and segmental duplications.
- The CUPs identified for the full-genome data were the same regardless of the conversion tool used, indicating that they are determined by the chain files.
- Pre-excluding SNVs at these CUPs prior to conversion results in SNVs that are stable to conversion.

## Supplementary Material

genomeBuildConversion_Ormond_Supplementary_2021-04-09_bbab069

## Data Availability

The data underlying this article (including BED files for the CUPs as well as the source code for the algorithm to generate them) are available at https://github.com/cathaloruaidh/genomeBuildConversion/.

## Notes

**Cathal Ormond** is a PhD Student in the Neuropsychiatric Genetics Research Group in the Department of Psychiatry, Trinity College Dublin.

**Niamh M. Ryan** is a Postdoctoral Research Fellow in the Neuropsychiatric Genetics Research Group in the Department of Psychiatry, Trinity College Dublin.

**Aiden Corvin** is a Professor in the Neuropsychiatric Genetics Research Group and the Head of the Department of Psychiatry, Trinity College Dublin.

**Elizabeth A. Heron** is an Assistant Professor in Biostatistical Genomics in the Neuropsychiatric Genetics Research Group in the Department of Psychiatry, Trinity College Dublin.
