# Supplementary material for: Converting single nucleotide variants between genome builds: from cautionary tale to solution
Source: Brief Bioinform. 2021 Apr 5;22(5):bbab069. doi: 10.1093/bib/bbab069 (PMC8425424; doi:10.1093/bib/bbab069)
Supplement: genomeBuildConversion_Ormond_Supplementary_2021-04-09_bbab069 [file genomebuildconversion_ormond_supplementary_2021-04-09_bbab069.docx]

**Supplementary Information for**

**Title**

Converting single nucleotide variants between genome builds: from cautionary tale to solution.

**Authors**

Cathal Ormond^1^, Niamh M Ryan^1^, Aiden Corvin^1^, Elizabeth A Heron^1^

**Affiliations**

^1:^ Neuropsychiatric Genetics Research Group, Department of Psychiatry, Trinity College Dublin, Ireland

# Supplementary Figures

Supplementary Figure S1: Venn diagrams displaying the proportion overlap of the assembly annotation sets for A) GRCh37 and B) GRCh38. Gap: gaps in the assembly; ContigDiff: differences in contigs between builds; SegDup: segmental duplications.

| A) GRCh37 | B) GRCh38 |
| --- | --- |
| 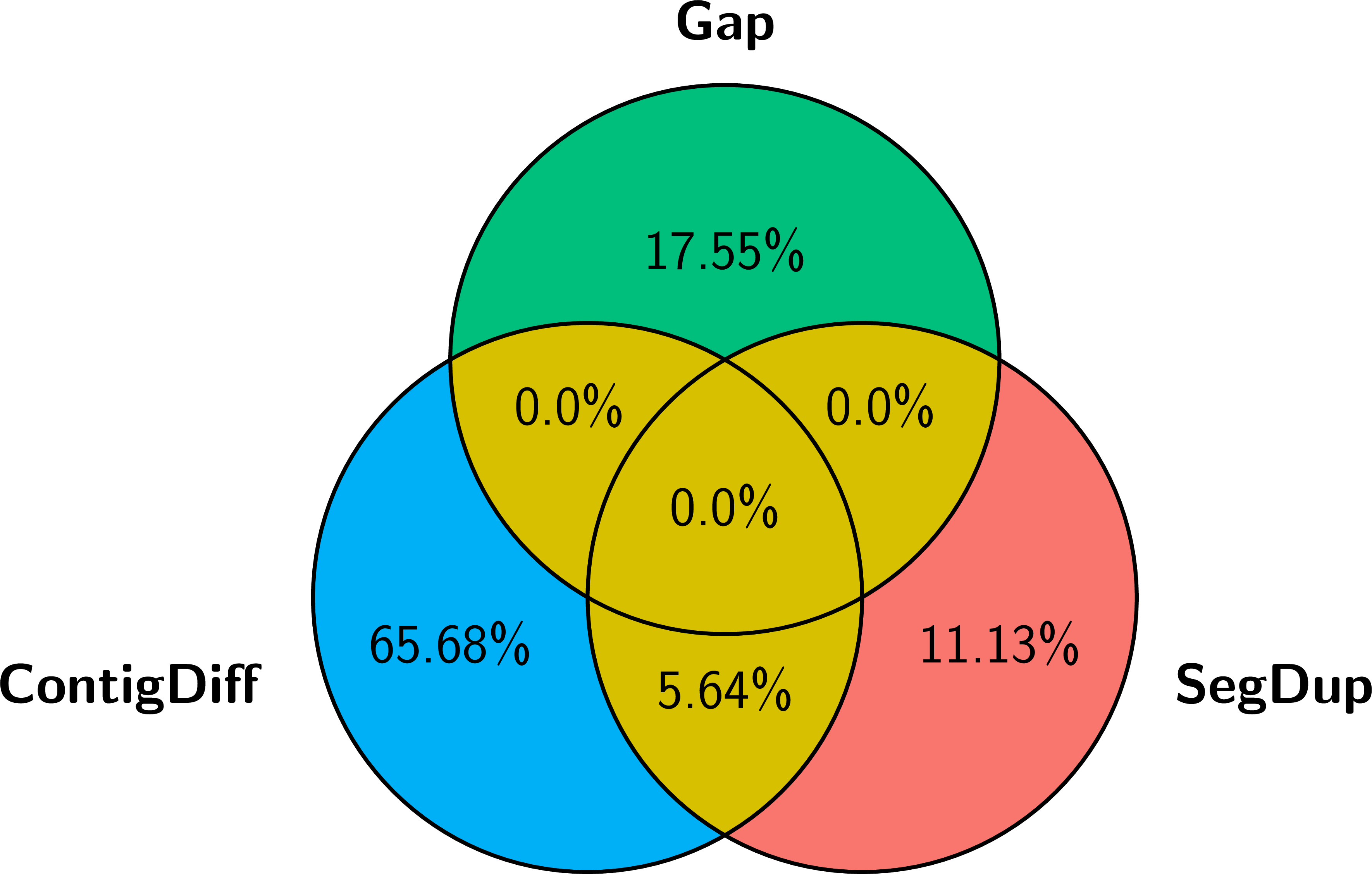 | 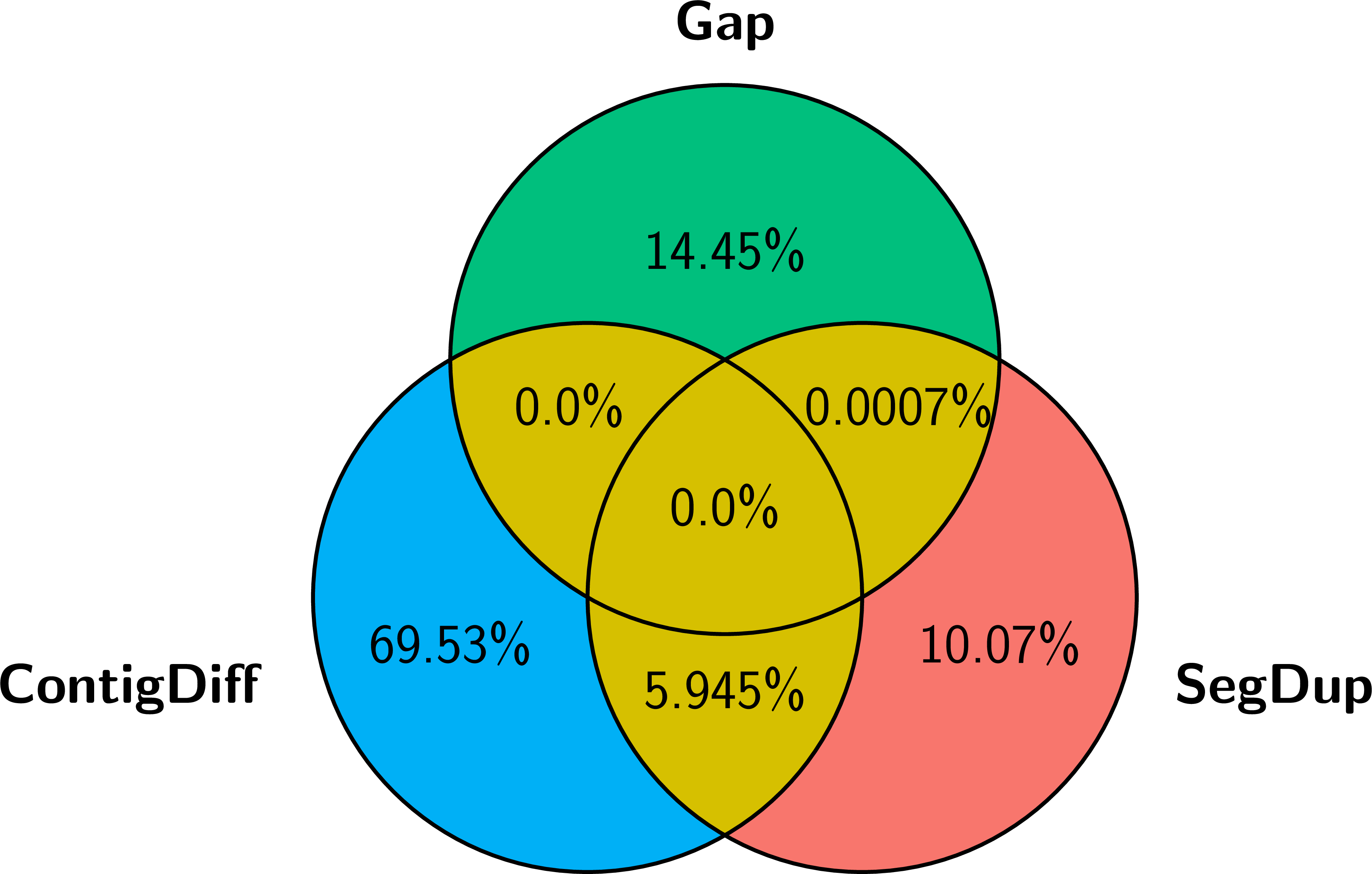 |

# Supplementary Tables

Supplementary Table S1: Details of the stable and conversion-unstable positions for the full-genome data of GRCh37 and GRCh38, including the number of base-pairs (bp) for each category, the proportion of the genome covered, the number of contiguous segments in each category (Regions), and the mean, standard deviation (SD), median and range of the number of base-pairs per region.

| **Source** | **Category** | **37 to 38 (bp)** | **% of**  **Source** | **38 to 37 (bp)** | **% of Source** | **Regions** | **Mean (bp)** | **SD (bp)** | **Median (bp)** | **Range (bp)** |
| --- | --- | --- | --- | --- | --- | --- | --- | --- | --- | --- |
| GRCh37 | All | 3,095,677,412 | 100.00 | 2,859,470,792 | 92.37 | - | - | - | - | - |
|  | Reject_1 | 234,712,067 | 7.58 | - | - | 29,442 | 7,972.0 | 341,047.00 | 1 | (1; 30,000,000) |
|  | CHR_Jump_1 | 1,494,553 | 0.05 | - | - | 1,570 | 951.9 | 9,862.04 | 70 | (1; 201,709) |
|  | Reject_2 | - | - | 100,180 | 0.00 | 9,706 | 10.3 | 43.81 | 2 | (1; 2,570) |
|  | CHR_Jump_2 | - | - | 799,922 | 0.03 | 1,229 | 650.9 | 1,814.30 | 143 | (1; 36,132) |
|  | POS_Jump | - | - | 8,907,439 | 0.29 | 20,684 | 430.6 | 1,774.62 | 111 | (1; 119,819) |
|  | Stable | 2,859,470,792 | 92.37 | 2,849,663,251 | 92.05 | - | - | - | - | - |
|  | Novel CUPs | - | - | 11,302,094 | 0.37 | 17,815 | 634.4 | 4,242.42 | 100 | (1; 201,709) |
|  |  | **38 to 37 (bp)** | **% of Source** | **37 to 38 (bp)** | **% of Source** |  |  |  |  |  |
| GRCh38 | All | 3,088,269,832 | 100.00 | 2,862,067,878 | 92.68 | - | - | - | - | - |
|  | Reject_1 | 218,510,733 | 7.08 | - | - | 72,554 | 3,012.0 | 184,422.00 | 13 | (1; 30,000,000) |
|  | CHR_Jump_1 | 7,691,221 | 0.25 | - | - | 26,382 | 291.5 | 2,426.65 | 96 | (1; 186,858) |
|  | Reject_2 | - | - | 73,770 | 0.00 | 6,650 | 11.1 | 66.93 | 2 | (1; 2,460) |
|  | CHR_Jump_2 | - | - | 292,083 | 0.01 | 827 | 353.2 | 1,017.47 | 61 | (1; 11,998) |
|  | POS_Jump | - | - | 12,038,774 | 0.39 | 38,042 | 316.5 | 987.39 | 113 | (1; 48,061) |
|  | Stable | 2,862,067,878 | 92.68 | 2,849,663,251 | 92.27 | - | - | - | - | - |
|  | Novel CUPs | - | - | 20,095,848 | 0.65 | 60,560 | 331.8 | 1,918.21 | 108 | (1; 186,858) |

Supplementary Table S2: The proportion of each of the assembly annotation sets with the conversion unstable-position (CUP) categories. Gap: gaps in the assembly; ContigDiff: differences in contigs between builds; SegDup: segmental duplications; Multi: multiple assembly annotation sets; Sum; the sum of the previous four sets; Other: no assembly annotation sets.

| **Source** | **Category** | **Gap** | **ContigDiff** | **SegDup** | **Multi** | **Sum** | **Other** |
| --- | --- | --- | --- | --- | --- | --- | --- |
| GRCh37 | CHR_Jump_1 | 0.00000000 | 0.11486800 | 0.49189100 | 0.36833700 | 0.97509600 | 0.02490400 |
| GRCh37 | CHR_Jump_2 | 0.00000000 | 0.00198644 | 0.00383662 | 0.99417200 | 0.99999500 | 0.00000500 |
| GRCh37 | POS_Jump | 0.00000000 | 0.02165490 | 0.14548200 | 0.83278300 | 0.99992000 | 0.00008000 |
| GRCh37 | Reject_1 | 0.69073900 | 0.00072158 | 0.00004152 | 0.00078961 | 0.69229200 | 0.30770800 |
| GRCh37 | Reject_2 | 0.00000000 | 0.21918500 | 0.09873230 | 0.67144100 | 0.98935900 | 0.01064100 |
| GRCh38 | CHR_Jump_1 | 0.00000000 | 0.26123800 | 0.10304900 | 0.61645700 | 0.98074400 | 0.01925600 |
| GRCh38 | CHR_Jump_2 | 0.00000000 | 0.46379600 | 0.00333124 | 0.53287200 | 1.00000000 | 0.00000000 |
| GRCh38 | POS_Jump | 0.00000000 | 0.19667600 | 0.08564660 | 0.71764900 | 0.99997100 | 0.00002900 |
| GRCh38 | Reject_1 | 0.68922500 | 0.28655600 | 0.00006294 | 0.02415410 | 0.99999700 | 0.00000300 |
| GRCh38 | Reject_2 | 0.00000000 | 0.32395300 | 0.01726990 | 0.65842500 | 0.99964800 | 0.00035200 |

Supplementary Table S3: Counts of single nucleotide variants in each of the conversion-unstable position (CUP) categories using ***liftOver*** to convert, for both builds, both samples (NA12877 and NA12878), both data sources (BED and VCF data), and for original and filtered data. A) GRCh37; B) GRCh38

A)

|  | **NA12877** | | | | **NA12878** | | | |
| --- | --- | --- | --- | --- | --- | --- | --- | --- |
|  | **BED** | | **VCF** | | **BED** | | **VCF** | |
|  | **Original** | **Filtered** | **Original** | **Filtered** | **Original** | **Filtered** | **Original** | **Filtered** |
| **All** | 3,518,008 | 3,512,449 | 3,518,008 | 3,512,449 | 3,523,638 | 3,518,229 | 3,523,638 | 3,518,229 |
| **Reject_1** | 4,944 | 4,944 | 4,944 | 4,944 | 4,947 | 4,947 | 4,947 | 4,947 |
| **CHR_Jump_1** | 1,182 | 0 | 1,070 | 0 | 874 | 0 | 761 | 0 |
| **Mismatch_1** | - | - | 20,552 | 19,925 | - | - | 20,533 | 19,976 |
| **Reject_2** | 293 | 0 | 166 | 0 | 262 | 0 | 178 | 0 |
| **CHR_Jump_2** | 171 | 0 | 134 | 0 | 238 | 0 | 192 | 0 |
| **POS_Jump** | 3,913 | 0 | 3,428 | 0 | 4,035 | 0 | 3,593 | 0 |
| **Mismatch_2** | - | - | 134 | 0 | - | - | 128 | 0 |
| **Novel CUP** | 5,559 | 0 | 4,798 | 0 | 5,409 | 0 | 4,724 | 0 |
| **Stable** | 3,507,505 | 3,507,505 | 3,487,580 | 3,487,580 | 3,513,282 | 3,513,282 | 3,493,306 | 3,493,306 |

B)

|  | **NA12877** | | | | **NA12878** | | | |
| --- | --- | --- | --- | --- | --- | --- | --- | --- |
|  | **BED** | | **VCF** | | **BED** | | **VCF** | |
|  | **Original** | **Filtered** | **Original** | **Filtered** | **Original** | **Filtered** | **Original** | **Filtered** |
| **All** | 3,576,396 | 3,571,274 | 3,576,396 | 3,571,274 | 3,594,064 | 3,588,396 | 3,594,064 | 3,588,396 |
| **Reject_1** | 23,902 | 23,902 | 23,902 | 23,902 | 25,852 | 25,852 | 25,852 | 25,852 |
| **CHR_Jump_1** | 1,828 | 0 | 1,551 | 0 | 1,979 | 0 | 1,706 | 0 |
| **Mismatch_1** | - | - | 16,219 | 15,273 | - | - | 16,772 | 15,741 |
| **Reject_2** | 180 | 0 | 88 | 0 | 187 | 0 | 94 | 0 |
| **CHR_Jump_2** | 308 | 0 | 158 | 0 | 428 | 0 | 181 | 0 |
| **POS_Jump** | 2,806 | 0 | 2,320 | 0 | 3,074 | 0 | 2,571 | 0 |
| **Mismatch_2** | - | - | 59 | 0 | - | - | 85 | 0 |
| **Novel CUP** | 5,122 | 0 | 4,117 | 0 | 5,668 | 0 | 4,552 | 0 |
| **Stable** | 3,547,372 | 3,547,372 | 3,532,099 | 3,532,099 | 3,562,544 | 3,562,544 | 3,546,803 | 3,546,803 |

Supplementary Table S4: Counts of single nucleotide variants in each of the conversion-unstable position (CUP) categories using ***CrossMap*** to convert, for both builds, both samples (NA12877 and NA12878), both data sources (BED and VCF data), and for original and filtered data. A) GRCh37; B) GRCh38

A)

|  | **NA12877** | | | | **NA12878** | | | |
| --- | --- | --- | --- | --- | --- | --- | --- | --- |
|  | **BED** | | **VCF** | | **BED** | | **VCF** | |
|  | **Original** | **Filtered** | **Original** | **Filtered** | **Original** | **Filtered** | **Original** | **Filtered** |
| **All** | 3,518,008 | 3,512,449 | 3,518,008 | 3,512,449 | 3,523,638 | 3,518,229 | 3,523,638 | 3,518,229 |
| **Reject_1** | 4,944 | 4,944 | 4,944 | 4,944 | 4,947 | 4,947 | 4,947 | 4,947 |
| **CHR_Jump_1** | 1,182 | 0 | 1,070 | 0 | 874 | 0 | 761 | 0 |
| **Mismatch_1** | - | - | 20,534 | 19,914 | - | - | 20,510 | 19,959 |
| **Reject_2** | 293 | 0 | 166 | 0 | 262 | 0 | 178 | 0 |
| **CHR_Jump_2** | 171 | 0 | 135 | 0 | 238 | 0 | 192 | 0 |
| **POS_Jump** | 3,913 | 0 | 3,438 | 0 | 4,035 | 0 | 3,604 | 0 |
| **Mismatch_2** | - | - | 130 | 0 | - | - | 123 | 0 |
| **Novel CUP** | 5,559 | 0 | 4,809 | 0 | 5,409 | 0 | 4,735 | 0 |
| **Stable** | 3,507,505 | 3,507,505 | 3,487,591 | 3,487,591 | 3,513,282 | 3,513,282 | 3,493,323 | 3,493,323 |

B)

|  | **NA12877** | | | | **NA12878** | | | |
| --- | --- | --- | --- | --- | --- | --- | --- | --- |
|  | **BED** | | **VCF** | | **BED** | | **VCF** | |
|  | **Original** | **Filtered** | **Original** | **Filtered** | **Original** | **Filtered** | **Original** | **Filtered** |
| **All** | 3,576,396 | 3,571,274 | 3,576,396 | 3,571,274 | 3,594,064 | 3,588,396 | 3,594,064 | 3,588,396 |
| **Reject_1** | 23,902 | 23,902 | 23,902 | 23,902 | 25,852 | 25,852 | 25,852 | 25,852 |
| **CHR_Jump_1** | 1,828 | 0 | 1,556 | 0 | 1,979 | 0 | 1,710 | 0 |
| **Mismatch_1** | - | - | 16,186 | 15,254 | - | - | 16,740 | 15,726 |
| **Reject_2** | 180 | 0 | 88 | 0 | 187 | 0 | 95 | 0 |
| **CHR_Jump_2** | 308 | 0 | 158 | 0 | 428 | 0 | 181 | 0 |
| **POS_Jump** | 2,806 | 0 | 2,331 | 0 | 3,074 | 0 | 2,587 | 0 |
| **Mismatch_2** | - | - | 57 | 0 | - | - | 81 | 0 |
| **Novel CUP** | 5,122 | 0 | 4,133 | 0 | 5,668 | 0 | 4,573 | 0 |
| **Stable** | 3,547,372 | 3,547,372 | 3,532,118 | 3,532,118 | 3,562,544 | 3,562,544 | 3,546,818 | 3,546,818 |

Supplementary Table S5: Discordance rates between converted data and aligned data for position and genotype, for both WGS samples, both conversion tools, and comparing original or filtered data.

| **Conversion** | **Sample** | **Category** | **Tool** | **Position Discordance** | **Genotype Discordance** | **Combined Discordance** |
| --- | --- | --- | --- | --- | --- | --- |
| GRCh38 to GRCh37 | NA12877 | filtered | liftOver | 2.9754% | 0.0010% | 2.9764% |
| GRCh38 to GRCh37 | NA12877 | filtered | CrossMap | 2.9759% | 0.0010% | 2.9769% |
| GRCh38 to GRCh37 | NA12877 | original | liftOver | 3.0694% | 0.0012% | 3.0706% |
| GRCh38 to GRCh37 | NA12877 | original | CrossMap | 3.0703% | 0.0012% | 3.0715% |
| GRCh38 to GRCh37 | NA12878 | filtered | liftOver | 2.9654% | 0.0011% | 2.9665% |
| GRCh38 to GRCh37 | NA12878 | filtered | CrossMap | 2.9658% | 0.0011% | 2.9669% |
| GRCh38 to GRCh37 | NA12878 | original | liftOver | 3.0691% | 0.0012% | 3.0703% |
| GRCh38 to GRCh37 | NA12878 | original | CrossMap | 3.0700% | 0.0012% | 3.0712% |
| GRCh37 to GRCh38 | NA12877 | filtered | liftOver | 1.7369% | 0.0010% | 1.7379% |
| GRCh37 to GRCh38 | NA12877 | filtered | CrossMap | 1.7371% | 0.0010% | 1.7381% |
| GRCh37 to GRCh38 | NA12877 | original | liftOver | 1.8119% | 0.0012% | 1.8131% |
| GRCh37 to GRCh38 | NA12877 | original | CrossMap | 1.8123% | 0.0012% | 1.8135% |
| GRCh37 to GRCh38 | NA12878 | filtered | liftOver | 1.4794% | 0.0011% | 1.4805% |
| GRCh37 to GRCh38 | NA12878 | filtered | CrossMap | 1.4798% | 0.0011% | 1.4809% |
| GRCh37 to GRCh38 | NA12878 | original | liftOver | 1.5544% | 0.0013% | 1.5557% |
| GRCh37 to GRCh38 | NA12878 | original | CrossMap | 1.5550% | 0.0013% | 1.5563% |
